# Clinical advances in immunotherapy for immune-mediated glomerular diseases

**DOI:** 10.1007/s10238-023-01218-7

**Published:** 2023-10-27

**Authors:** Bihui Tang, Xiao Yang

**Affiliations:** Department of Nephrology, Union Hospital, Tongji Medical College, Huazhong University of Science and Technology, Wuhan, 430022 Hubei China

**Keywords:** Immune-mediated glomerular diseases, Immunotherapy, Clinical advances

## Abstract

**Background and objective:**

Due to the suboptimal therapeutic efficacy and potential adverse effects associated with traditional immunosuppressive medications, there has been an increasing emphasis on the development and utilization of immunotherapies. This paper aims to provide clinicians with valuable insights for selecting appropriate therapeutic approaches and contribute to the development of novel immunotherapeutic drugs.

**Main body:**

This paper categorizes the immunotherapeutic drugs that are used for the treatment of immune-mediated glomerular diseases into three groups: immunotherapies targeting antigen-presenting cells (anti-CD80), immunotherapies targeting T/B cells (anti-CD20, anti-CD22, BAFF and APRIL inhibitors, CD40-CD40L inhibitors, proteasome inhibitors, Syk inhibitors, and Btk inhibitors), and immunotherapies targeting the complement system (C5 inhibitors, C5a/C5aR inhibitors, C3 inhibitors, MASP2 inhibitors, factor B inhibitors, and factor D inhibitors). The article then provides a comprehensive overview of advances related to these immunotherapeutic drugs in clinical research.

**Conclusion:**

Certain immunotherapeutic drugs, such as rituximab, belimumab, and eculizumab, have exhibited notable efficacy in treating specific immune-mediated glomerular diseases, thereby providing novel therapeutic approaches for patients. Nonetheless, the efficacy of numerous immunotherapeutic drugs remains to be substantiated.

## Background

The human immune system plays a crucial role in the occurrence and progression of various renal diseases, including immune-mediated glomerular diseases. When the immune system is abnormally activated due to multiple stimuli, it triggers the activation of both innate and adaptive immunity, leading to glomerular injury. Immune-mediated glomerular diseases can cause a series of symptoms, such as albuminuria, hematuria, and renal dysfunction. Without active intervention, the majority of cases will progress to end-stage renal disease. The current treatment options for immune-mediated glomerular diseases primarily involve supportive care and the use of traditional immunosuppressive agents such as glucocorticoids (GCs), alkylating agents, calcineurin inhibitors (CNIs), and mycophenolate mofetil (MMF). However, these therapeutic measures are nonspecific, and their efficacy is sometimes not ideal. Moreover, long-term use of traditional immunosuppressive agents tends to cause many serious side effects [1]. Fortunately, immunotherapeutic drugs such as rituximab have given patients new hope as the pathogenesis of immune-mediated glomerular diseases has begun to be better understood. Since rituximab was successfully used in the treatment of renal diseases, various immunotherapeutic drugs have emerged and are being investigated for the treatment of immune-mediated glomerular diseases. Therefore, this article aims to offer clinicians valuable insights for selecting appropriate therapeutic approaches and contribute to the development of novel immunotherapeutic drugs.

## Immune response processes

**Fig. 1:**
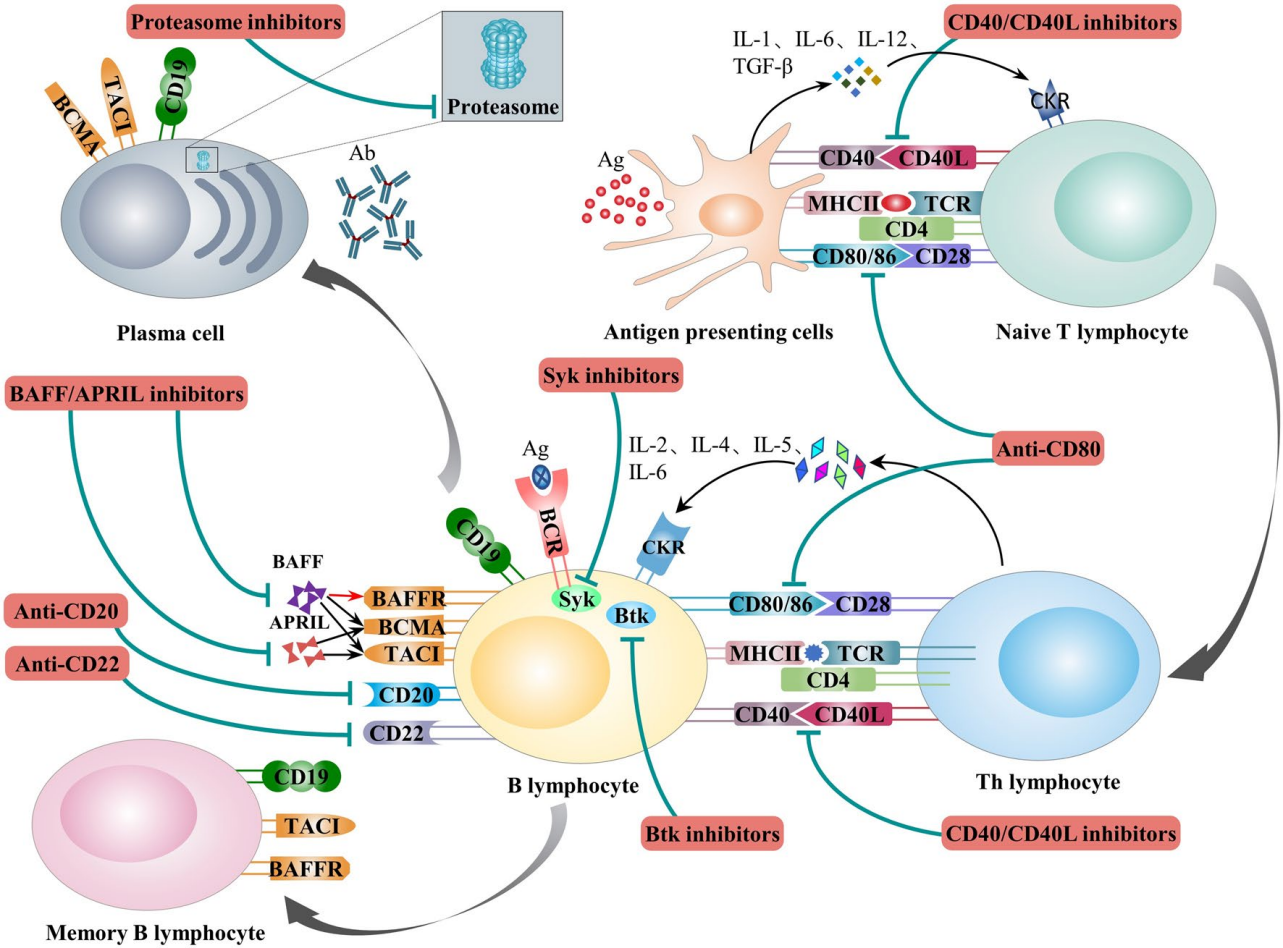
Immune response process and targets of immunotherapies in T/B cells and antigen-presenting cells

Immune-mediated glomerular diseases are primarily caused by immune abnormalities, and factors involved in adaptive immunity, including cellular and humoral immunity, interact with innate immune factors to mediate kidney injury [2]. In cellular immunity, T cell antigen receptors (TCRs) recognize and bind to the specific peptide-major histocompatibility complex (pMHC) presented by antigen presenting cells (APCs) to produce the first signal, while CD80/CD86 on the surface of APCs binds to CD28 on the surface of T cells to provide the second signal to T cells. Simultaneously, the cytokines secreted by activated APCs provide the third signal to T cells, which along with the first and second signals, induces T cell activation, proliferation, and differentiation. Activated T cells express the costimulatory molecule CD40L, which can bind to CD40 on the surface of APCs, thus inducing APC activation and the expression of more costimulatory molecules. These costimulatory molecules bind to corresponding receptors on the T-cell surface to further promote T cell proliferation. Activated CD4^+^ T cells differentiate into helper T (Th) lymphocyte subsets with different functions. Recent studies have demonstrated the close associations among the Th1, Th2, and Th17 cell subsets and the pathogenesis of immune-mediated glomerular diseases [3]. In addition to cellular immunity, humoral immunity also plays a crucial role in mediating the occurrence and progression of immune-mediated glomerular diseases. In humoral immunity, B-cell antigen receptors (BCRs) recognize and bind specific antigenic epitopes to initiate the first signal for B-cell activation; then activate T cells highly express CD40L, which binds to CD40 on the surface of B cells to provide the second signal. A variety of cytokines secreted by T cells provide the third signal, and the three signals together induce B-cell activation, proliferation, and differentiation, with some B cells differentiating into plasma cells, which in turn secrete pathogenic antibodies that cause kidney injury, and other B cells differentiating into memory B cells, which may exert lasting pathogenic effects (Fig. 1). Furthermore, innate immune components such as neutrophils, macrophages, natural killer cells and the complement system also play essential roles in the occurrence and development of immune-mediated glomerular diseases [4, 5].

Immunotherapy is a new type of therapy that suppresses or activates the immune response involved in immune-mediated glomerular diseases by targeting specific components of the immune response. In this way, immunotherapy effectively prevents the kidney damage caused by a persistent immune response. This paper divides immunotherapeutic drugs into three categories based on their targets: (1) immunotherapies targeting APCs, (2) immunotherapies targeting T/B cells, and (3) immunotherapies targeting the complement system.

## Immunotherapies targeting APCs

### Anti-CD80

CD80, also known as B7-1, is a transmembrane protein typically expressed on the surface of APCs and B cells. On APCs, CD80 binds to the receptor CD28 on T cells, providing an indispensable signal for T cell activation. CD80 can also bind to the receptor CTLA-4 on T cells, limiting T-cell activation and proliferation [6]. Abatacept, a recombinant fusion protein targeting CD80, consists of the extracellular structural domain of human CTLA4 and part of the Fc structural domain of human IgG1. This CTLA4 immunoglobulin molecule competes with CD28 to bind CD80/86 on the APC surface, inhibiting T-cell activation and T-cell-dependent B-cell activation by blocking the interactions between CD28 and CD80/86. At the same time, abatacept decreases the levels of various cytokines, including TNF-*α*, IL-2, IL-4, IL-5, IL-6, and IFN-*γ* [7]. Abatacept can also inhibit the formation of T follicular helper cells and induce the transformation of naive T cells into regulatory T cells (Tregs) [8]. Thus, abatacept has a potential therapeutic role in immune-mediated glomerular diseases.

Abatacept is being studied for the treatment of lupus nephritis (LN). Although a multicenter phase 2/3 RCT of abatacept combined with MMF and GCs failed to reach the primary endpoint, the time to confirmed complete response, it did show that treatment with abatacept was associated with improved levels of anti-dsDNA antibodies, complement, and urinary protein, which supported further evaluation of abatacept for the treatment of LN [9]. A subsequent study of abatacept combined with cyclophosphamide (CTX) still failed to meet the primary endpoint [10]. Meanwhile, a phase 3 clinical trial of abatacept on a background of MMF (ALLURE) also showed no statistically significant difference in efficacy and safety between the abatacept group and control group. However, abatacept-treated patients had more rapid improvements in proteinuria, leading to an earlier, sustained complete response [11]. Thus, more research is required to clarify the effect of abatacept on LN patients. Additionally, a case report documented the successful use of abatacept for the treatment of five patients with focal segmental glomerulosclerosis (FSGS) (four patients with post-transplant recurrent FSGS and one with primary FSGS); partial or complete remission of proteinuria was induced in these patients [12], which provided support for subsequent clinical trials of abatacept for the treatment of FSGS. A phase 2 pilot RCT evaluating the safety and efficacy of abatacept in the treatment of severe albuminuria caused by FSGS and minimal change nephropathy (MCD) has been completed. However, the relevant data have not yet been made public. It is believed that these data will give us a better understanding of the effects of abatacept on FSGS.

## Immunotherapies targeting T/B cells

### Anti-CD20

CD20 is a non-glycosylated protein expressed on the surface of B cells and a general surface marker expressed by most B cells from pre-B lymphocytes onwards, but its expression is absent in plasma mother cells and plasma cells. CD20 participates in BCR signal transduction and affects the phosphorylation of BCR-linked kinases and proteins, such as LYN, SYK, GAB1, and ERK, which are necessary for effective BCR signal transduction. CD20 deficiency can decrease the number of circulating memory B cells, antibody class switching, and IgG levels [13]. Therefore, CD20 has become a significant target for treating immune-mediated glomerular diseases.

Rituximab, the most widely used anti-CD20 monoclonal antibody, is the first biologic agent for the treatment of kidney disease. It works by targeting and eliminating CD20^+^ B cells through various mechanisms, including antibody-dependent cellular cytotoxicity (ADCC), antibody-dependent cell-mediated phagocytosis (ADCP), and complement-dependent cytotoxicity (CDC). Rituximab has been widely used in the treatment of various immune-mediated glomerular diseases, such as membranous nephropathy (MN), FSGS, MCD, LN, IgA nephropathy (IgAN), and anti-neutrophil cytoplasmic antibody-associated vasculitides (AAVs) [14]. Newer-generation anti-CD20 monoclonal antibodies based on rituximab, including ocrelizumab, ofatumumab, and Obinutuzumab, have been developed. Overall, rituximab is the most commonly used anti-CD20 monoclonal antibody. Rituximab is usually chosen first in clinical trials conducted to explore the efficacy of anti-CD20 monoclonal antibodies. When rituximab proved to be ineffective, next-generation anti-CD20 monoclonal antibodies were considered as a new option. However, there is currently a lack of clinical trials comparing the efficacy of rituximab and next-generation anti-CD20 monoclonal antibodies in the treatment of diseases for which anti-CD20 monoclonal antibodies have established effects, although some trials of other diseases, such as lymphocytic leukemia and follicular lymphoma, have confirmed the superiority of the next-generation anti-CD20 monoclonal antibodies over rituximab [15, 16]. Because next-generation anti-CD20 monoclonal antibodies are humanized, doctors may consider replacing rituximab with the new anti-CD20 monoclonal antibodies for patients with a rituximab allergy.

Rituximab is particularly prominent in the treatment of MN, and its efficacy and safety have been demonstrated by several clinical trials [17–20]. Therefore, it has been used extensively around the globe and is presently recommended as the first-line treatment for idiopathic membranous nephropathy in the Kidney Disease: Improving Global Outcomes (KDIGO) guidelines [21]. Many patients with MN have greatly benefited from anti-CD20 monoclonal antibodies, especially those who did not respond to traditional immunosuppressants.

However, there are still some contradictory reports on rituximab in the treatment of IgAN. Although some case reports have reported its potential therapeutic effects [22], a multicenter RCT revealed that rituximab was unable to significantly lower urinary protein levels or protect renal function in patients with IgAN and failed to lower serum levels of galactose-deficient IgA1 and anti-galactose-deficient IgA1 antibodies [23]. Therefore, RCTs with a large sample size are required to further prove its efficacy. Rituximab and renin–angiotensin–aldosterone system (RASS) inhibitors are being used to treat IgAN in a multicenter RCT (RITA study, NCT04525729) in China.

The effectiveness of rituximab in the treatment of FSGS and MCD lacks strong support from high-quality evidence. Several retrospective studies have shown that rituximab can effectively decrease the frequency of relapse and the need for traditional immunosuppressants, which suggests that rituximab is a potentially effective therapeutic agent [24–28]. A prospective study (the NEMO study) also reached a similar positive result [29], while another small prospective study indicated that rituximab had little impact in the treatment of FSGS patients [30]. Overall, all of these studies are either retrospective or nonrandomized controlled trials, which cannot provide sufficient evidence for the efficacy of rituximab in the treatment of FSGS and MCD. Therefore, to further validate the precise therapeutic effects of rituximab, large-scale prospective multicenter RCTs are needed. Three RCTs (TURING (EudraCT:2018–004611-50), RIFIREINS (NCT03970577), and NCT03298698) are now in progress and may provide valuable data.

Multiple observational studies suggested that rituximab could be an effective and safe alternative for treating LN [31–34], but a multicenter phase 3 RCT revealed that rituximab yielded no clinical improvement in LN patients [35]. Some studies have demonstrated that rituximab-induced B-cell depletion can increase the levels of B-cell activating factor (BAFF) and trigger the production of autoantibodies, which could accelerate the progression of the disease [36]. Based on this research theory, rituximab combined with belimumab, which targets BAFF, has been investigated for the treatment of LN. However, compared to rituximab alone, this combination therapy did not exhibit significantly increased clinical efficacy [37]. Obinutuzumab, a humanized type II anti-CD20 monoclonal antibody, has a distinct mode of binding to the CD20 antigen and a greater affinity for the Fc*γ* receptor on effector cells, which gives obinutuzumab several advantages over rituximab in depleting B cells. A multicenter phase 2 RCT showed improvements in overall renal remission and serological indexes reflecting disease activity in patients with proliferative LN who were treated with obinutuzumab [38]. A multicenter double-blind phase 3 RCT is currently being performed to further evaluate the efficacy of obinutuzumab for the treatment of LN.

Rituximab has been shown in numerous RCTs to have efficacy similar to that of cyclophosphamide and azathioprine in the treatment of AAVs [39–41]. Consequently, rituximab has been recommended as a standard treatment for inducing and maintaining remission of AAVs in the KDIGO guidelines and a preferred option for reinduction therapy in patients with relapsing AAVs [21]. Both the FDA and EMA have approved AAVs as an indication for rituximab. Next-generation CD20 monoclonal antibodies are also being used, but thorough research demonstrating their therapeutic advantages over rituximab is still lacking [42].

Due to the rarity of anti-glomerular basement membrane (anti-GBM) disease, there has been no RCT to verify the therapeutic effect of anti-CD20 monoclonal antibodies. Some case reports and small retrospective studies have reported that rituximab has a clear effect [43–46], which means that it has potential as a novel treatment alternative for anti-GBM disease.

### Anti-CD22

CD22 is a type I transmembrane sialic acid glycoprotein belonging to the immunoglobulin superfamily. It is specifically expressed in B lymphocytes. The expression level of CD22 varies during the development of B lymphocytes. Initially, it is expressed at a low level in the cytoplasm of pro-B cells and pre-B cells. However, it translocates to the cell surface as B cells mature and becomes highly expressed in mature B cells. Eventually, it is absent on the surface of plasma mother cells and plasma cells [47]. As the inhibitory coreceptor of BCR, CD22 plays a key role in the development and survival of B cells and is thought to be a crucial component of humoral immune system regulation [48].

Epratuzumab is a humanized anti-CD22 monoclonal antibody that can downregulate BCR signaling by binding to CD22 and inducing CD22 phosphorylation, leading to cell death [49]. It also downregulates CD19, CD79*β*, and CD21 on the B-cell surface, raising the BCR threshold and further reducing the BCR signal [50]. Moreover, epratuzumab can regulate immunity by inhibiting the production of pro-inflammatory cytokines such as TNF and IL-6 without affecting the production of anti-inflammatory cytokines such as IL-10 [51, 52]. Therefore, epratuzumab is a very important biological agent targeting B cells. It is currently mainly used to treat LN. Although an open-label trial and two phase 2 RCTs confirmed its efficacy and safety in treating systemic lupus erythematosus (SLE) [53–56], two phase 3 clinical trials lasting 48 weeks demonstrated that epratuzumab did not improve the remission rate of the disease [57]. Notably, epratuzumab decreased the number of peripheral B cells compared with that in the control group in the above clinical trials. Thus, further experimental investigation is needed to explore epratuzumab’s potential in treating immune-mediated glomerular diseases.

### Inhibitors of BAFF and a proliferation-inducing ligand (APRIL)

Both BAFF and APRIL are members of the tumor necrosis factor superfamily, and BAFF is also known as B lymphocyte-stimulating factor (BLyS). They share two receptors, namely, transmembrane activator and cyclophilin ligand interactor (TACI) and B-cell maturation antigen (BCMA). Furthermore, BAFF can bind to B-cell activating factor receptor (BAFFR). BAFF is mainly produced by neutrophils, monocytes, and macrophages [58]. It plays significant roles in B-cell survival, differentiation, maturation, antibody production and class switching [59]. In addition to its effect on B cells, studies have shown that BAFF can promote T cell activation, proliferation, and differentiation [60]. BAFF and APRIL are overexpressed in some autoimmune diseases, such as SLE, suggesting their potential involvement in the pathogenesis of these diseases [61].

When BAFF/APRIL inhibitors bind to BAFF/APRIL, they stop them from binding to their receptors, which reduces B-cell survival and prevents B cells from differentiating into plasma cells that produce antibodies. BAFF/APRIL inhibitors are currently utilized to treat various immune-mediated glomerular diseases, such as LN, MN, IgAN, and AAVs. Belimumab, a monoclonal antibody targeting BAFF, is the first approved BAFF inhibitor. Three multicenter double-blind phase 3 RCTs have confirmed its efficacy and safety in patients with SLE [62–64]. At the same time, a multicenter double-blind phase 3 RCT demonstrated that belimumab can protect renal function and minimize the risk of disease flares in LN patients [65]. Therefore, belimumab has been approved by the FDA and EMA to treat LN and SLE. It provides a new treatment option for LN patients and is being widely used in clinical settings, providing significant help to many LN patients. Another BAFF/APRIL inhibitor is atacicept, a chimeric recombinant fusion protein. It was found to reduce disease activity and severe exacerbations in SLE patients in a phase 2b clinical trial. The extension trial revealed that long-term use of atacicept is associated with long-lasting efficacy and a tolerable safety profile [66, 67]. Therefore, additional large-scale, well-designed clinical trials are needed to validate atacicept’s efficacy in the treatment of LN.

It is also important to note that further research is needed to clarify the efficacy of BAFF/APRIL inhibitors in patients with AAVs because a multicenter double-blind phase 3 RCT showed that the addition of belimumab during the remission maintenance phase of AAVs did not reduce the risk of relapse [68]. Furthermore, some studies found that BAFF/APRIL inhibitors could decrease the level of proteinuria in patients with IgAN and MN, which highlights the need for further clinical studies to explore the efficacy and safety of BAFF/APRIL inhibitors in patients with IgAN and MN [69, 70].

### CD40/CD40L inhibitors

CD40 is a transmembrane surface receptor that belongs to the tumor necrosis factor receptor superfamily and is expressed on various immune and nonimmune cells. Its ligand is CD40L. The interaction between CD40 and CD40L plays an important role in regulating humoral and cellular immunity [71, 72]. It is essential for B-cell proliferation and differentiation, the production of high-affinity antibodies, antibody class switching, costimulatory activity, and the activation of macrophages, dendritic cells and neutrophils. It can also regulate Th1 differentiation, CD8^+^ cytotoxic T lymphocyte (CTL) activation, and memory CTL maintenance [73]. According to some studies, abnormal CD40-CD40L signal transduction may contribute to the initiation and maintenance of the autoimmune response [74]. Therefore, CD40/CD40L inhibitors have the potential to treat autoimmune disorders by inhibiting the CD40 signaling pathway and inactivating immune cells.

CD40/CD40L inhibitors have primarily been researched for treating LN. Numerous animal studies have revealed that CD40/CD40L inhibitors can successfully reduce proteinuria and lengthen survival in mice with LN, as well as delay and reduce illness flares in mice prone to LN. Additionally, a study found a strong link between the pathogenesis of LN and the CD40-CD40L signaling pathway [75]. Ruplizumab (BG9588), a first-generation CD40/CD40L inhibitor, was the subject of a multicenter open-label phase 1/2 trial for the treatment of LN, but the trial was prematurely stopped due to thromboembolic events. Preliminary data from the trial showed that ruplizumab can reduce anti-dsDNA antibody titres and decrease haematuria, indicating its therapeutic potential [76]. However, a multicenter double-blind phase 2 RCT found no significant difference between IDEC-131, another first-generation CD40/CD40L inhibitor, and placebo in patients with SLE [77]. And all clinical trials of IDEC-131 were stopped due to thromboembolic events in a trial for the treatment of Crohn’s disease [78]. Later, researchers developed second-generation CD40/CD40L inhibitors, such as dapirolizumab pegol (CDP7657), BI 655064, and iscalimab (CFZ533), to prevent thromboembolic events. A multicenter double-blind phase 1 RCT demonstrated that dapirolizumab pegol was safe in SLE patients and improved the condition of patients [79]. However, a subsequent multicenter double-blind phase 2 RCT was unsuccessful in achieving the primary end point [80]. Therefore, additional research is needed to explore the clinical effects of dapirolizumab pegol. Meanwhile, the results of a multicenter double-blind phase 2 RCT to assess BI655064 in the treatment of LN (NCT02770170) have not yet been announced, and another study to assess the effects of iscalimab (CFZ533) (NCT03610516) is now being conducted. Data from these trials will improve our greater understanding of the therapeutic effects of CD40/CD40L inhibitors in LN.

### Proteasome inhibitors

The proteasome–ubiquitin system, a tightly regulated protein degradation system, plays critical roles in many important biological processes, including MHC-mediated antigen presentation, cytokine and cell cycle regulation, and apoptosis. By controlling the degradation of key proteins, this system significantly influences the development and progression of autoimmune diseases [81]. Proteasome inhibitors can cause many misfolded proteins to accumulate in the plasma cell endoplasmic reticulum, thus activating the unfolded protein response signaling pathway and eventually leading to plasma cell cycle arrest and apoptosis [82]. Plasma cell apoptosis causes a reduction in pathogenic autoimmune antibody levels. In addition, studies have demonstrated that proteasome inhibitors can selectively target pro-inflammatory cytokines and their receptors, as well as interfere with intracellular signaling pathways within pro-inflammatory immune effector cells, which ultimately inhibits autoimmune responses [81]. Hence, the proteasome is a promising therapeutic target for the treatment of immune-mediated glomerular diseases.

Bortezomib, a proteasome inhibitor, has been widely used in clinical practice and is currently being investigated for the treatment of LN and IgAN. Several case reports and retrospective studies have provided evidence for the use of bortezomib in the treatment of refractory LN [83–86]. Two small prospective noncontrolled studies further confirmed that bortezomib was able to reduce proteinuria, protect renal function, and decrease autoantibody titres in patients with refractory LN [87, 88]. A multicenter double-blind phase 2 RCT conducted in Japan demonstrated improvements in the symptoms of bortezomib-treated patients, such as joint inflammation and rash, but the bortezomib group did not exhibit decreased levels of anti-dsDNA antibodies compared with the placebo group and was prone to side effects, such as fever, headache, facial swelling, liver dysfunction, and thrombocytopenia [89]. Therefore, bortezomib should only be used as an unconventional treatment for LN patients, and care should be taken to avoid its side effects. Meanwhile, a small prospective uncontrolled study verified that bortezomib could reduce proteinuria in IgAN patients who possessed an Oxford classification T score of 0 and retained normal renal function [90], but further confirmation from randomized controlled studies is still lacking. There is also evidence from multiple case reports indicating that bortezomib has potential for the treatment of AAVs and MN [91–93]. Consequently, it is recommended that further RCTs be conducted to investigate the efficacy of bortezomib in these diseases.

### Spleen tyrosine kinase (Syk) inhibitors

Syk is a 72 kDa cytoplasmic nonreceptor protein tyrosine kinase expressed in various cell types, such as B cells, immature T cells, mast cells, neutrophils, macrophages, and platelets. The transduction of B-cell receptor (BCR) and Fc receptor initiation signals is heavily reliant on Syk [94]. Syk-mediated BCR signaling is indispensable for B-cell maturation and survival, and Syk-deficient B cells stagnate at the pre-B-cell stage [95]. Recent preclinical and clinical studies have confirmed that Syk is involved in the pathogenesis of proliferative glomerulonephritis, including anti-GBM disease, LN, and IgAN [96]. Inhibiting Syk activity may prevent the production of pathogenic autoantibodies and inhibit the production of pro-inflammatory cytokines in immune-mediated glomerular diseases [97]. Thus, Syk has emerged as a promising therapeutic target for the treatment of immune-mediated glomerular diseases.

Fostamatinib, a small molecule Syk inhibitor, is currently under investigation for the treatment of IgAN. A multicenter double-blind phase 2 RCT (NCT02112838) was conducted to evaluate the efficacy and safety of fostamatinib in the treatment of IgAN, but the results have not yet been published. The preliminary data showed that fostamatinib failed to reduce proteinuria in IgAN patients, but a subgroup analysis indicated that fostamatinib may benefit IgAN patients who exhibit high levels of proteinuria at baseline [98]. This finding provides favorable evidence supporting further clinical trials.

### Bruton tyrosine kinase (Btk) inhibitors

Btk is a cytoplasmic nonreceptor protein tyrosine kinase in the Tec family that is expressed in a variety of immune cells, including monocytes, macrophages, basophils, mast cells, and B cells. Btk is an essential component of the immune response, as it regulates signals downstream of the BCR, the Fc receptor, and Toll-like receptors [99, 100]. Btk inhibitors can affect the survival of B cells by inhibiting BCR signals, thus reducing the production of autoantibodies [101]. Furthermore, it can regulate signal transduction mediated by Toll-like and Fc receptors to mitigate the damage caused by autoantibody deposition in tissue [102, 103]. It can also influence other immune cells, such as by influencing the production of cytokines and inflammatory mediators [99], neutrophil recruitment [104], the maturation and function of dendritic cells [105], the activation of NK cells [106], and the function of T cells[107]. Considering Btk’s significant influence on diverse cellular signaling pathways and its involvement in both adaptive and innate immunity, it is regarded as a crucial target for the treatment of autoimmune diseases.

To date, Btk inhibitors have mainly been investigated for the treatment of LN. Several animal experiments have been conducted to illustrate the efficacy of Btk inhibitors in reducing autoantibody titres, alleviating proteinuria, and delaying renal injury in mice with LN [101, 108–110], which provides a theoretical basis for the initiation of clinical trials. Unfortunately, the therapeutic efficacy observed in animal trials has not been consistently replicated in clinical trials. Two multicenter double-blind phase 2 RCTs showed that Btk inhibitors (evobrutinib, fenebrutinib) had no significant impact compared with the placebo, and the trials failed to reach the primary endpoint [111, 112]. Several other clinical trials evaluating the efficacy of multiple Btk inhibitors for the treatment of LN are currently underway, and hopefully, positive results will be reported in the future.

## Immunotherapies targeting the complement system

**Fig. 2:**
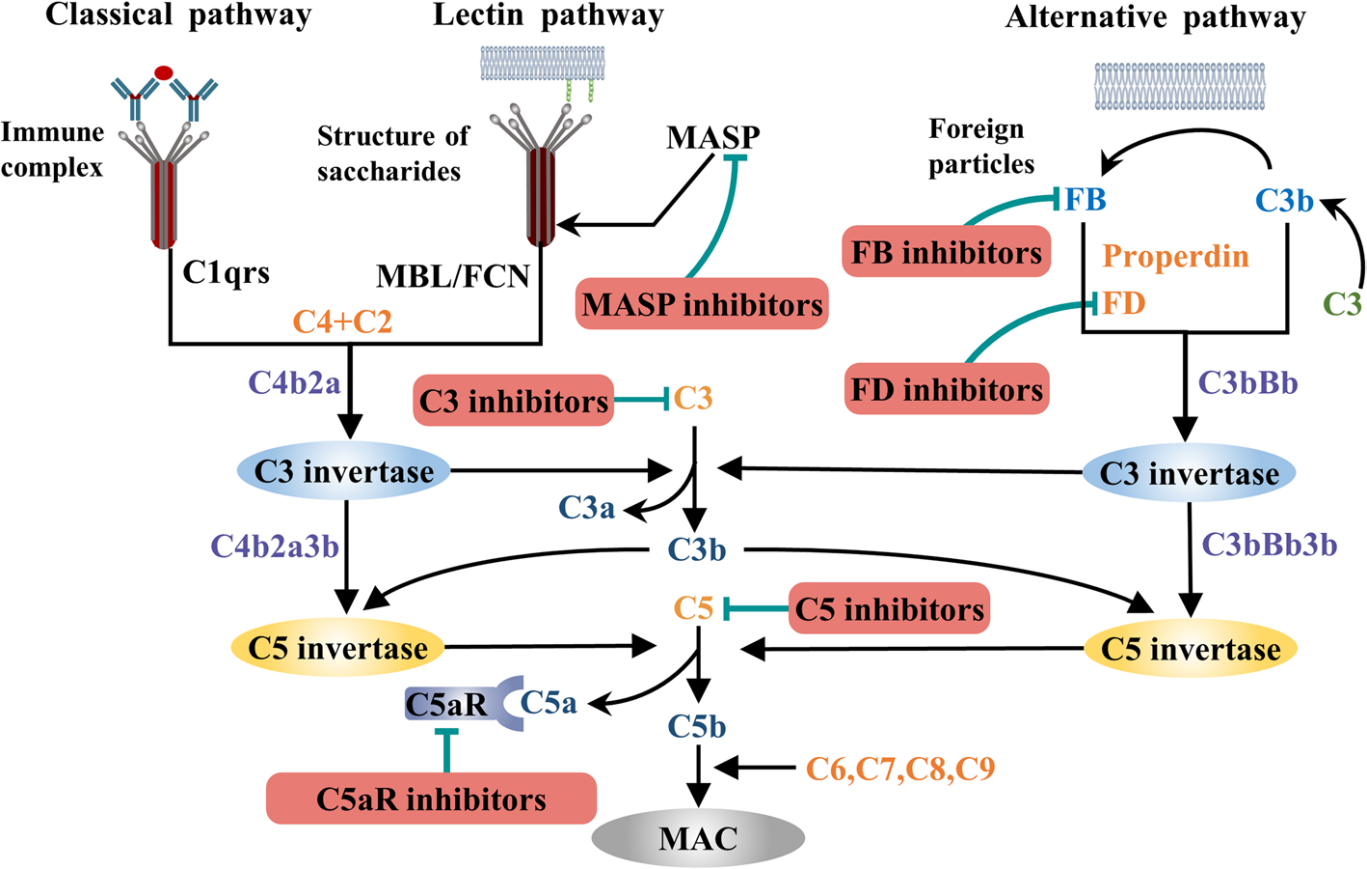
Immunotherapies targeting the complement system. *MBL* Mannan-Binding Lectin; *FCN* Ficolin; *MASP* MBL-Associated Serine Protease; *FB* Factor B; *FD* Factor D; *C5aR* C5a Receptor; *MAC* Membrane Attack Complex

The complement system is not only the central component of innate immunity but also the bridge between innate immunity and the adaptive immune response. It can be activated in three ways: the classical pathway, lectin pathway and alternative pathway. In the classical pathway, the Fc fragment of the immune complex binds to Clq and then activates C1r, C1s, C4, C2 and C3 to form C3 invertase. In the lectin pathway, mannose binds lectin or fibrinogen to recognize the sugar structure on the surface of the pathogen and then activates mannan-binding lectin-associated serine protease (MASP) to form C3 invertase. In the alternative pathway, factor B binds to C3b on the surface of microorganisms and forms C3 invertase with the help of factor D and properdin. C3 invertase is further activated to form C5 invertase, which cleaves C5 into C5a and C5b. Eventually, C5b binds to C6-C9 to form the membrane attack complex (MAC), which mediates cytolysis (Fig. 2). Incorrect activation or dysregulation of the complement cascade can lead to the destruction of host cells. Complement activation has been confirmed to be involved in the occurrence and progression of many renal diseases, such as atypical hemolytic uremic syndrome (aHUS), C3 glomerulonephritis (C3GN), AAVs, IgAN, LN, and MN. Therefore, the inhibition or regulation of the complement system has been considered a promising treatment strategy for immune-mediated glomerular diseases for many years. To date, a variety of drugs have been developed, but their clinical application is still limited.

### C5 inhibitors

C5 inhibitors, including eculizumab, ravulizumab and crovalimab, are the most widely used biological agents targeting the complement system and block the formation of the MAC by inhibiting the cleavage of C5 into C5a and C5b. C5 inhibitors are mainly used in the treatment of aHUS and C3GN and may be beneficial in the treatment of IgAN and LN.

Eculizumab, a humanized anti-C5 monoclonal antibody, is the first complement inhibitory drug approved by the FDA in the United States. To date, two multicenter prospective open-label phase 2 clinical trials have confirmed eculizumab’s efficacy in patients with aHUS, and early intervention is associated with greater clinical benefits [113]. Long-term follow-up data over the next two years demonstrated the sustained therapeutic efficacy of eculizumab in the treatment of aHUS [114]. Another multicenter prospective clinical trial reconfirmed the efficacy of eculizumab in the treatment of aHUS [115]. The safety of eculizumab has been fully confirmed by a 5-year observational study involving a total of 865 individuals [116]. Hence, there is expert consensus, and clinical guidelines recommend eculizumab as a first-line treatment for aHUS [117–119]. Four studies, including two retrospective studies and two prospective noncontrolled trials, confirmed that eculizumab is also effective in some patients with C3GN [120–123]. Nevertheless, importantly, the effect of eculizumab in patients with C3GN is limited because eculizumab is not able to directly block C3 cleavage product-mediated glomerular injury. Additionally, due to the rarity of C3GN, conducting large-scale RCTs to validate the therapeutic effect of eculizumab presents significant challenges. Therefore, eculizumab is currently used as the second-line treatment for C3GN. Meanwhile, it has been observed in two case reports that eculizumab may also be used in the treatment of IgAN [124, 125], but these reports are insufficient to confirm the efficacy of eculizumab, and further RCTs are needed.

Ravulizumab, another anti-C5 monoclonal antibody, was developed on the basis of eculizumab and shares the same mechanism, but it has a longer half-life. A multicenter prospective open-label phase 3 clinical trial demonstrated that ravulizumab could effectively induce aHUS remission and improve renal function [126]. The mid-term report from the extended trial further confirmed the efficacy and safety of ravulizumab in the treatment of aHUS, with further clinical improvements achieved with long-term use [127]. Consequently, ravulizumab has been recommended for the treatment of aHUS in many countries. To date, there is no clear evidence to suggest that eculizumab and ravulizumab have differences in the treatment of aHUS. Both drugs have been approved by the FDA and EMA for the treatment of aHUS. Nevertheless, because ravulizumab has a longer half-life and a longer injection interval, it can enhance patient compliance, improve patient quality of life and satisfaction, and decrease medical costs. In addition, clinical trials of ravulizumab for the treatment of IgAN and LN are underway [128].

### C5a receptor (C5aR)/C5a inhibitors

Avacopan is a new type of oral C5a receptor-specific inhibitor that can block the binding of C5a and C5aR without affecting the production of C5b or the MAC, reducing the risk of side effects such as infection. Recent studies have revealed the importance of the interaction between C5a and C5aR in the development of AAVs. This discovery highlights the potential to use C5aR/C5a inhibitors to treat AAVs [129–131]. Two multicenter double-blind phase 2 RCTs have shown that adding avacopan to the standard treatment for AAVs can more quickly control renal inflammation and reduce renal damage, with the expectation of decreasing or completely avoiding the use of GCs [132, 133]. Based on these two trials, a multicenter double-blind phase 3 RCT was conducted to evaluate the use of avacopan in the treatment of AAVs; this trial indicated that avacopan can replace GCs as the standard treatment for AAVs, thus avoiding the side effects caused by GCs [134]. Avacopan has been approved by the FDA and EMA for the treatment of AAVs. Apart from its use for treating AAVs, avacopan has also been explored for the treatment of IgAN. An open-label phase 2 trial demonstrated the positive effects and safety of avacopan [135]. However, this trial has certain limitations, such as a small sample size, short treatment time, and no control group. Therefore, further large-scale RCTs are necessary to validate the therapeutic effect of avacopan on IgAN. In addition to avacopan, there is a monoclonal antibody directly targeting C5a, vilobelimab, which also blocks the interaction of C5a with C5aR and is therefore also considered to be useful for the treatment of AAVs. Two multicenter double-blind phase 2 RCTs (NCT03895801, NCT03712345) are evaluating its efficacy and safety [136], but specific results from these trials have not yet been released.

### Other complement inhibitors

Pegcetacoplan is a C3 inhibitor that not only binds to C3 and C3b to inhibit their activation but also inhibits the activity of invertase containing C3b subunits, including C3 and C5 invertase associated with the alternative pathway and C5 invertase associated with the classical pathway [137]. By acting directly at the level of C3, pegcetacoplan is anticipated to exhibit efficacy in the treatment of C3GN. Currently, several phase 2 and 3 clinical trials are evaluating the efficacy and safety of pegcetacoplan in the treatment of C3GN.

Narsoplimab (OMS-721) is an anti-MASP2 monoclonal antibody that blocks the initiation of the lectin pathway by inhibiting MASP2, thereby affecting the production of the MAC. An interim report from a multicenter phase 2 clinical trial suggests that narsoplimab has the potential to reduce albuminuria and maintain a stable eGFR in high-risk patients with advanced IgAN [138]. There is also an ongoing multicenter double-blind phase 3 RCT evaluating the efficacy and safety of narsoplimab in the treatment of IgAN. In addition, several clinical trials are underway to assess narsoplimab in the treatment of LN, MN, C3GN, and aHUS patients.

Iptacopan (LNP-023) is an oral specific inhibitor of factor B that blocks the initiation of the alternative pathway by inhibiting C3 convertase, thereby disrupting the generation of downstream C5 convertase and subsequently affecting the onset of the terminal cascade of the complement system. Preclinical studies have shown that the inhibition of factor B can prevent the activation of the complement system in patients with C3GN [139], which provides a theoretical basis for the utilization of factor B inhibitors in the treatment of C3GN. A phase 2 study showed that iptacopan could significantly reduce albuminuria and improve renal function in patients with C3GN [140]. Another multicenter double-blind phase 3 RCT evaluating the efficacy and safety of iptacopan in the treatment of C3GN is ongoing [141]. Additionally, several trials are underway to explore the safety and efficacy of iptacopan in the treatment of IgAN, aHUS, and LN.

Danicopan is a factor D-specific inhibitor that can inhibit the initiation of the alternative pathway. Therefore, danicopan is expected to become a prospective therapeutic intervention for individuals diagnosed with aHUS and C3GN [142]. It is hoped that relevant clinical studies can be carried out in the future.

## Conclusions and future perspectives

The utilization of immunotherapy is a notable advancement in the treatment of immune-mediated glomerular diseases. This development introduces a novel concept for treating immune-mediated glomerular diseases, with remarkable outcomes. Certain immunotherapeutic drugs, such as rituximab, belimumab, eculizumab, and ravulizumab, have exhibited notable efficacy in the treatment of specific immune-mediated glomerular diseases, thereby offering novel therapeutic approaches for patients. However, numerous obstacles to the clinical application of immunotherapy persist. Many drugs have not been investigated in large RCTs or clinical trials or have been demonstrated to be unable to achieve the anticipated therapeutic effects. Consequently, there is insufficient high-quality evidence to ascertain their long-term efficacy and safety or determine the optimal treatment regimen, which restricts their widespread utilization in clinical settings. Given the uncertain efficacy of immunotherapeutic drugs in clinical applications, many studies need to be conducted to further explore the pathogenesis of all kinds of immune-mediated glomerular diseases and comprehensively elucidate the mechanisms of action of different immunotherapeutic drugs. This will help researchers select appropriate immunotherapeutic drugs for treatment. Furthermore, it is also imperative to thoroughly analyze patient indicators to identify the pertinent factors that influence the efficacy of immunotherapeutic drugs. Simultaneously, continued efforts should be made to enhance the development and design of diverse immunotherapeutic drugs, aiming to minimize adverse effects, improve therapeutic efficacy, and optimize drug delivery strategies, thereby facilitating their improved utilization in the clinic.

This article reviews only the more mature immunotherapeutic drugs targeting T/B cells, APCs and the complement system (Table 1). Although other immunotherapeutic drugs, such as immunosuppressive agents that target IL-2, IL-17, and IFN, have been investigated for the treatment of immune-mediated glomerular diseases in the clinic, these drugs are not discussed in detail within this article. Currently, the number of immunotherapeutic drugs that can be used to treat immune-mediated glomerular diseases is expanding. Various biological agents that were originally developed for treating other diseases are being actively explored for their potential application in patients with immune-mediated glomerular diseases. In addition, many researchers are developing novel biological agents, as well as conducting diverse clinical trials. We suspect that in the near future, with a deeper understanding of the pathogenesis of immune-mediated glomerular diseases and a large amount of data obtained in various clinical trials, immunotherapies will enable precise and effective treatment of patients with immune-mediated glomerular diseases.

**Table 1:** Summary of the application of immunotherapeutic drugs in immune-mediated glomerular diseases

Drugs	Diseases	Evidence	Efficacy	Security
*Anti-CD80*				
Abatacept	LN	Phase 3 clinical trial proved ineffective	Further investigation	Well tolerated
	FSGS	The case report proved effective	Insufficient data	Insufficient data
*Anti-CD20*				
Rituximab	MN	Phase 3 clinical trial proved effective	Established	Well tolerated
	IgAN	Multicenter RCT proved ineffective	Further investigation	Well tolerated
	FSGS, MCD	Prospective study proved effective	Further investigation	Well tolerated
	LN	Phase 3 clinical trial proved ineffective	Further investigation	Well tolerated
	AAVs	Phase 3 clinical trial proved effective	Established	Well tolerated
	Anti-GBM disease	Case reports and retrospective trials proved effective	Insufficient data	Insufficient data
Obinutuzumab	LN	Phase 2 clinical trial proved effective	Further investigation	Well tolerated
*Anti-CD22*				
Epratuzumab	LN	Phase 3 clinical trial proved ineffective	Further investigation	Well tolerated
*BAFF/APRIL inhibitors*				
Belimumab	LN	Phase 3 clinical trial proved effective	Established	Well tolerated
	AAVs	Phase 3 clinical trial proved ineffective	Further investigation	Well tolerated
	MN	Prospective clinical trial proved effective	Further investigation	Well tolerated
Atacicept	LN	Phase 2 clinical trial proved effective	Further investigation	Well tolerated
	IgAN	Phase 2 clinical trial proved effective	Further investigation	Well tolerated
*CD40/CD40L inhibitors*				
Ruplizumab	LN	Phase 1/2 trial proved effective	Further investigation	Thromboembolic events
IDEC-131	LN	Phase 2 trial proved ineffective	Further investigation	Thromboembolic events
Dapirolizumab pegol	LN	Phase 2 trial proved ineffective	Further investigation	Well tolerated
*Proteasome inhibitors*				
Bortezomib	LN	Phase 2 trial proved ineffective	Further investigation	Prone to side effects
	IgAN	Prospective uncontrolled study proved effective	Further investigation	Insufficient data
	MN	The case report proved effective	Insufficient data	Insufficient data
	AAVs	The case report proved effective	Insufficient data	Insufficient data
*Syk inhibitors*				
Fostamatinib	IgAN	Phase 2 trial proved ineffective	Further investigation	Well tolerated
*Btk inhibitors*				
Evobrutinib	LN	Phase 2 trial proved ineffective	Further investigation	Well tolerated
Fenebrutinib	LN	Phase 2 trial proved ineffective	Further investigation	Well tolerated
*C5 inhibitors*				
Eculizumab	aHUS	Phase 2 trial proved effective	Established	Well tolerated
	C3GN	Prospective uncontrolled study proved effective	Further investigation	Well tolerated
	IgAN	The case report proved effective	Insufficient data	Insufficient data
Ravulizumab	aHUS	Phase 3 trial proved effective	Established	Well tolerated
*C5aR/C5a inhibitors*				
Avacopan	AAVs	Phase 3 trial proved effective	Established	Well tolerated
	IgAN	Phase 2 trial proved effective	Insufficient data	Insufficient data
Vilobelimab	AAVs	Data unpublished	No data	No data
*C3 inhibitors*				
Pegcetacoplan	C3GN	No data	No data	No data
*MASP2 inhibitors*				
Narsoplimab	IgAN	Phase 2 trial proved effective	Further investigation	Well tolerated
*Factor B inhibitors*				
Iptacopan	C3GN	Phase 2 trial proved effective	Further investigation	Well tolerated
*Factor D inhibitors*				
Danicopan	C3GN, aHUS	No data	No data	No data

*LN* Lupus Nephritis; *FSGS* Focal Segmental Glomerulosclerosis; *MN* Membranous Nephropathy; *IgAN* IgA Nephropathy; *MCD* Minimal Change nephropathy; *AAVs* Anti-Neutrophil Cytoplasmic Antibody-Associated Vasculitides; *Anti-GBM disease* Anti-Glomerular Basement Membrane Disease; *aHUS* Atypical Hemolytic Uremic Syndrome; *C3GN* C3 Glomerulonephritis
